# Revolutionizing Urban Mobility: IoT-Enhanced Autonomous Parking Solutions with Transfer Learning for Smart Cities

**DOI:** 10.3390/s23218753

**Published:** 2023-10-27

**Authors:** Qaiser Abbas, Gulzar Ahmad, Tahir Alyas, Turki Alghamdi, Yazed Alsaawy, Ali Alzahrani

**Affiliations:** 1 Faculty of Computer and Information Systems, Islamic University of Madinah, Madinah 42351, Saudi Arabia; qabbas@iu.edu.sa (Q.A.); dr.turki2@iu.edu.sa (T.A.); yalsaawy@iu.edu.sa (Y.A.); a.alzahrani@iu.edu.sa (A.A.); 2 Department of Computer Science & IT, University of Sargodha, Sargodha 40100, Pakistan; 3 Department of Computer Science, University of South Asia, Lahore 54000, Pakistan; gulzar.ahmad@usa.edu.pk; 4 Department of Computer Science, Lahore Garrison University, Lahore 54000, Pakistan

**Keywords:** cloud computing, IoT, smart city, performance, secure data management, modeling

## Abstract

Smart cities have emerged as a specialized domain encompassing various technologies, transitioning from civil engineering to technology-driven solutions. The accelerated development of technologies, such as the Internet of Things (IoT), software-defined networks (SDN), 5G, artificial intelligence, cognitive science, and analytics, has played a crucial role in providing solutions for smart cities. Smart cities heavily rely on devices, ad hoc networks, and cloud computing to integrate and streamline various activities towards common goals. However, the complexity arising from multiple cloud service providers offering myriad services necessitates a stable and coherent platform for sustainable operations. The Smart City Operational Platform Ecology (SCOPE) model has been developed to address the growing demands, and incorporates machine learning, cognitive correlates, ecosystem management, and security. SCOPE provides an ecosystem that establishes a balance for achieving sustainability and progress. In the context of smart cities, Internet of Things (IoT) devices play a significant role in enabling automation and data capture. This research paper focuses on a specific module of SCOPE, which deals with data processing and learning mechanisms for object identification in smart cities. Specifically, it presents a car parking system that utilizes smart identification techniques to identify vacant slots. The learning controller in SCOPE employs a two-tier approach, and utilizes two different models, namely Alex Net and YOLO, to ensure procedural stability and improvement.

## 1. Introduction

The concept of a smart city has emerged with multiple new challenges and opportunities in IT governance, development, security, and emerging technologies. Digital systems for smart cities are becoming a new breed of software with real-time updates, connectivity, and functionality. It is, therefore, highly desirable to formulate such frameworks and models that support the concept and functionality of a smart city [1].

A smart city is a well-defined and new concept that many institutions and researchers focus on nowadays. Inappropriate parking of vehicles at parking spots may lead to a deadlock situation for the rest of the other vehicles. It is an issue for the entire world to handle such a situation. The problem is in locating an appropriate parking spot in minimal time and by using fewer resources and without wasting time, ensuring safe and secure parking. By using the Internet of Things, artificial intelligence, and other communication devices, this problem can be resolved [2].

Internet of Things (IoT) and artificial intelligence are the major research areas used to solve challenges related to transportation and other smart city problems. IoT refers to the services of interconnected devices, people, networks, and other valuable things that are provided with radio frequency identification (RFID). Publishing data onto the cloud requires no human-to-human interaction. These IoT-enabled devices use multiple communication, networking, and data-linking protocols.

**Figure 1:**
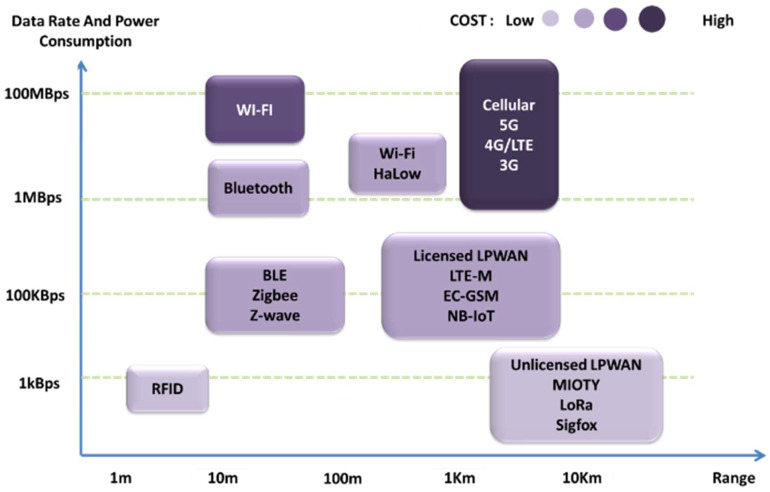
Comparison among LPWA networks and many other connectivity technologies.

The rapid development of the Internet of Things (IoT) enables ubiquitous connectivity among various machines through wireless communication, significantly impacting people’s daily life in many domains, such as smart cities, smart homes, garbage monitoring, smart parking, smart transportation, etc. The surrounding information is sensed by IoT devices and shared with people for efficient services and among themselves. In smart parking, short-term wireless networks are used. Multiple techniques such as Bluetooth, ZigBee, Wi-Fi, long-term evolution (LTE), etc. achieve efficient communication [3]. These methods can provide reliable communications and high-speed data transmissions between IoT devices. The Low Power Wide Area (LPWA) networks, which employ a novel wireless protocol, are also very famous in the field of transmission in the current era due to their long-range communication at low power. They offer high energy efficiency, low power consumption, and high coverage capabilities. Figure 1 compares LPWA networks and many other connectivity technologies regarding power consumption, bandwidth, cost, and range.

Smart cities are complex ecosystems that involve many stakeholders (e.g., managed service providers, network operators, logistic centers), and must work together to achieve the best services. These ecosystems consist of the interactions between an environment and organisms. The emergence of multiple types of ecosystems can be seen in the world of online applications and electronics in which devices interact with one another. The ecosystems composed of multiple connected devices can do most of the data processing on their own and do not need any human intervention. Humans can interact with these devices to instruct the respective actor and set them up. Therefore, many smart infrastructures such as multi-sensor ecosystems are installed for collecting data on many roadsides [4]. 

Many other methods are used to collect data by using light detection and ranging (LiDAR), global positioning system (GPS), variable messaging signs (VMSs), and inertial measurement units (IMUs). The live information collected by these devices is then passed to the autonomous cloud gateway servers. One operational part of a city’s ecosystem are the smart parking systems. In smart cities, the notion of automatic parking systems is growing, and can enhance the comfort and safety of drivers. An automated parking lot system helps drivers to park their cars quickly and safely without any issues. 

Various machine learning (ML) and deep learning (DL) techniques have been used by many researchers to develop a novel system for better results. Moreover, these consist of machine learning models (such as support vector machines (svm), regression tree, random forest), time series models (such as AutoRegressive Integrated Moving Average (ARIMA) and AutoRegressive Moving Average (ARMA)), and ensemble techniques used to predict various domains (such as bank fraudulent detection, spam mail detection, future decision-making traffic congestion control). Artificial neural networks (ANN), which can learn independently, perform nonlinear fitting, etc., have also been used for the said problem. ML, DL, and ANN solve many problems in smart cities, such as smart buildings, roads, and parking. Other than these machine learning (ML) techniques, the queuing theory has also been used to predict the wait time before parking occupancy in parking lots, or in many other areas such as pattern recognition, speech recognition, signal processing, and control systems [5].

The development of smart cities is rapidly progressing, and is driven by the advancements in technology, data connectivity, and intelligent systems. One crucial aspect of this transformation is the effective utilization of wireless connections, which enable seamless communication and data exchange in urban environments. However, while the role of wireless connectivity is substantial, it is just one piece of the larger puzzle that constitutes a smart city’s infrastructure and functionality. A parking system in a smart city is a technologically advanced and integrated solution designed to optimize and streamline the management of parking spaces within urban environments. These systems leverage various technologies and data-driven approaches to address the challenges associated with parking, such as congestion, limited availability of parking spots, and inefficient space utilization.

The smart city concept represents a transformative approach to urban planning and management, which harnesses technology, data, and innovative solutions to address the complex challenges faced by modern urban centers. It aims to create more efficient, sustainable, and livable cities by integrating various aspects of urban life with cutting-edge technologies. 

### 1.1. Problem Statement

In the context of smart cities, urban congestion and limited parking spaces have become pressing challenges. Traditional parking management systems often fall short in efficiently utilizing available parking spaces and providing a seamless experience for drivers. To address this issue, this research focuses on developing an IoT-based autonomous parking scenario that leverages transfer learning techniques. The primary problem addressed within this research paper is the need for a more efficient and seamless parking system that not only optimizes parking space usage but also contributes to reducing urban traffic congestion and enhancing the quality of urban life. 

### 1.2. Research Motivations 

This research has formulated the following research motivations:

- Rapid urbanization has led to a surge in the number of vehicles on the road, resulting in chronic traffic congestion in many cities.
- The advancement of Internet of Things (IoT) technology presents an opportunity to revolutionize urban transportation and parking management.
- The potential for machine learning and transfer learning techniques to adapt and optimize autonomous parking systems across different smart city environments is a compelling avenue for exploration.

### 1.3. Significance of Our Study

This research aims to develop an innovative and efficient IoT-based autonomous parking system that enhances parking space utilization, reduces traffic congestion, and promotes the sustainable development of smart cities. This study’s significance lies in its potential to transform the way cities manage parking and urban mobility, ultimately leading to more sustainable, efficient, and user-centric smart cities. Alleviating traffic congestion is a critical concern in modern urban planning and transportation management. It refers to the efforts and strategies aimed at reducing or mitigating the congestion of vehicles on road networks during peak hours. Traffic congestion can lead to numerous negative consequences, including increased travel time, environmental pollution, fuel consumption, and stress, for commuters. The outcomes of our research have the potential to benefit not only the residents and visitors of smart cities but also the global urban community facing similar challenges.

### 1.4. Research Objectives 

The following objectives are defined for this research:

- Develop a robust module for an IoT-based autonomous parking system, dedicated to real-time data collection, analysis, and decision making. By leveraging advanced sensors and analytics, it will enable an automated detection of vacant and occupied parking spaces, improving the user experience and reducing the time spent searching for parking.
- Explore and apply transfer learning techniques to adapt the autonomous parking system to different smart city environments, promoting scalability and ease of deployment.
- Conduct extensive testing and evaluation of the developed system in real-world smart city environments to assess its effectiveness in optimizing parking space utilization and reducing traffic congestion.

## 2. Literature Review

IoT-based applications use recent developments in communication technology, artificial intelligence, sensor devices, ubiquitous computing, and wireless sensor networks (WSN). Cloud computing combined with the Internet of Things is speeding up the development of solutions that enable us to monitor traffic movement in smart cities. Many solutions have been devised to find parking spaces in smart cities to improve the quality of life. To provide comfort, smart parking systems help drivers to find available and free parking spaces, and they also keep in mind the number of free parking spaces available and the distance between them. Machine learning and deep learning methods have brought advancements and innovation in monitoring the mobility of vehicles in smart cities. Paidi et al. [6] suggested that combining computer vision and deep learning techniques can help find free parking lots. They discussed various techniques, technologies, and the applicability of sensors that can locate the availability of free parking space. Cai et al.’s [7] work was based on locating and measuring the traffic flow in parking lots with a novel vehicle filter based on deep learning techniques. The proposed system provided better accuracy as compared to other cheap industry benchmark systems. Vu and Huang [8] proposed a combination of spatial transform and deep contrastive network to conclude the investigation of parking space availability. The authors demonstrated that the technique was robust for parking displacements, distortion, variations in car sizes, effects of spatial variations, etc.

Zhang et al. [9] introduced a self-parking system based on deep learning techniques. In their proposed system, they marked the parking points in the image and then classified those points as occupied or free slots. Therefore, they developed an image database of parking slots that contains 12,165 images of outdoor and indoor parking slots. Chen et al. [10] reviewed the technologies of vision-based traffic semantic understanding in Intelligent Transportation Systems (ITSs).

Tekouabou et al. [11] introduced a combination of ensemble techniques and IoT devices to predict the number of free parking slots in smart cities and evaluated their system performance with the Birmingham parking dataset. They used the bagging ensemble technique and achieved a 94% prediction accuracy. Luo et al. [12] addressed the challenge of endogeneity in assessing the impact of Transport Infrastructure Connectivity (TIC) on local conflict resolution. They introduced novel evidence of TIC’s effects on conflict resolution through a natural experiment and the application of machine learning techniques, thus mitigating the concern of endogeneity. Orrie et al. [13] described wireless communication for the recommendations of the nearest parking spaces or reserve places with a GPS. After every 2 min, the system transmits information about the availability of free spaces. If no parking spaces are available, no actions are taken; on the other hand, within 2 km of their location, any user can reserve a place. The user receives a message on their smartphones with directions. If a car is parked in every slot, no action is performed; this application requires a Wi-Fi connection.

Karthi et al. [14] introduced a system in which they used a database and cloud to communicate to manage parking spaces in real time. The proposed system uses ultrasonic sensors that are placed on the ground, is connected via the internet, and has a mobile application for users to make reservations. Tabassum et al. [15] proposed, developed, and assessed four classifiers: multinomial Naive Bayes, decision tree, logistic regression, and random forest. The hyperparameters of the models were tuned, and it was concluded that the random forest outperformed the other classifiers with a 91.73% test and 100% training accuracy. The prediction systems based on neural networks have shown the importance of various factors, such as the day of the week, time of the day, temperature, and location. In contrast, traffic, events, rainfall, and vacation time play a secondary role. Therefore, it is crucial to develop and implement educational campaigns that target both drivers and pedestrians. Moreover, the differences between left- and right-hand driving and the potential risks associated with this should also be highlighted [16].

AlexNet was first proposed in 2012 by Alex Krizhevsky, and it is a simple, fundamental, and effective convolutional neural network that is mainly composed of different layers, namely convolutional layers, pooling layers, fully connected layers, and rectified linear unit (ReLU) layers [17]. It consists mainly of eight layers, in which five are convolutional, and three are fully connected. The first five convolutional layers extract the input features to generate convolved feature maps. In the pooling layer, average or max pooling operations are used on the convolved feature maps within the given neighborhood window to aggregate the information. AlexNet is booming due to its practical strategies, such as the dropout regularization technique and the ReLU non-linearity layer. ReLU can prevent overfitting and significantly accelerate the training phase, and it is a half-wave rectifier function. YOLO was introduced in 2016 by Joseph Redmon et al.; it performed well in object detection, and could detect objects in real time at 45 frames per second. There is also a smaller version of YOLO called Fast YOLO, which performs at 155 frames per second. In this study, we propose a mixed edge-based and cloud-based framework with the final goal of PM2.5 value prediction. In order to validate the proposed approach, we evaluate the quality of predictions using both original and preprocessed data on a real-world dataset from air quality sensors distributed in Calgary, Canada. [18] YOLO-V3 uses darknet as its backbone, and has a CNN with 53 layers; it is stacked with 53 more layers of CNNs, making the total convolutional layers equal to 106. Traffic flow prediction methods often depend on historical traffic data, including traffic volume and speed, but they may not be well suited for high-capacity expressways or periods of peak traffic congestion [19].

In this study, a driving simulator is employed to create driving scenarios and examine the driving performance of drivers with varying levels of experience in situations where they are faced with traffic rule violations performed by other road users. The experimental findings reveal that certain novice drivers disregard the positioning of their vehicles when encountering traffic violations, resulting in collisions with other road users. Furthermore, some novice drivers can only execute either steering or braking to evade collisions in these critical situations [20]. 

The obtained results show an average mean absolute percentage error improvement of 40.18% in the prediction accuracy by using the proposed preprocessing technique. Table 1 represents the year-wise key findings of different research focuses.

## 3. Solution Design and Implementation

### Conceptual Description of the Solution

**Figure 2:**
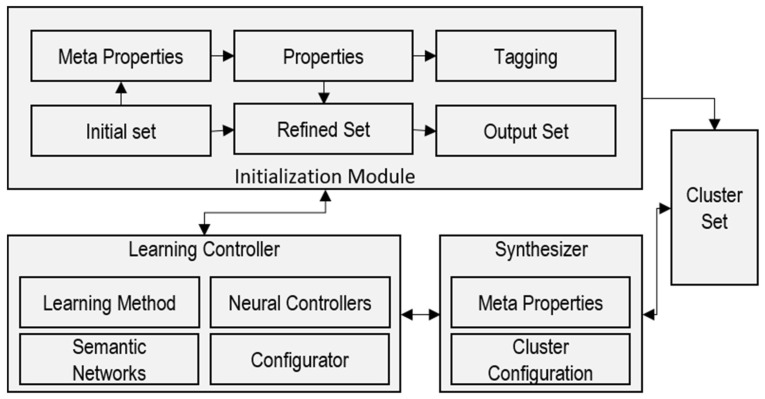
SCOPE preprocessing and learning.

Smart City Operation Platform Ecology (SCOPE) is a model which focuses on the provisioning of smart services and functions as a management system (Figure 2). It takes a smart city as an ecosystem with various inhabitants having specific needs and demands. One important component of this model is the controller that provides the learning algorithm and starts the system in terms of initialization. In this research, we are using the same module to identify parking. Three main modules used in this system include the initialization module, learning controller, and synthesizer. The initialization module serves as the starting point of the system. It is responsible for preparing and processing the raw input data. This module may involve tasks such as data preprocessing, which could include the cleaning, normalization, and transformation of the data. The initialization module provides preprocessing and tagging of datasets based on the properties required to formulate initial, refined, and output datasets. The learning controller is a crucial part of the system, as it manages the learning and decision-making processes. It likely includes machine learning algorithms or other AI techniques to analyze and learn from the processed data which it receives. The synthesizer is a separate component that plays a role in generating or synthesizing outputs or results. It might take the refined data from the learning controller and create meaningful outputs or solutions related to car parking.

**Figure 3:**
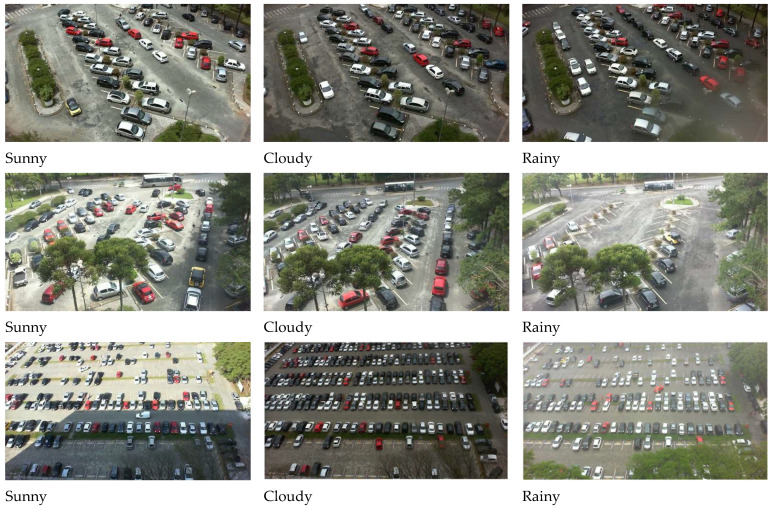
Various parking lots with different weather conditions.

There are 12,417 labeled images in the PKLot [26] parking dataset. The images in the dataset cover different kinds of climate conditions, such as rainy, sunny, and overcast preprocessing, in terms of the initial set, noise reduction, and elimination of other impurities to make a refined dataset for tagging and solution development periods (Figure 3). These images present distinct features because the dataset has different parking lots.

**Figure 4:**
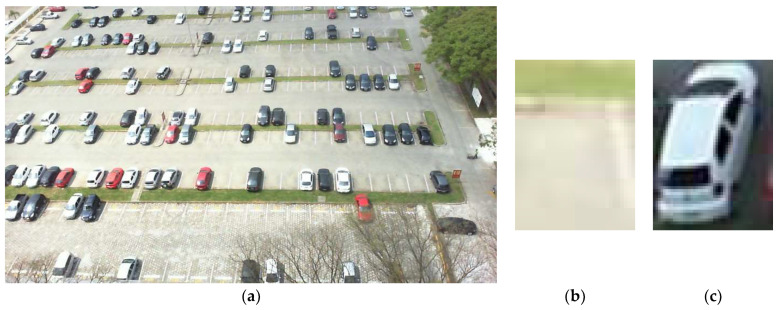
(**a**) Parking lot image, (**b**) empty sub image, and (**c**) occupied sub image.

This dataset is segmented into two classes: empty parking space class and the occupied space class. The total number of images after segmentation is 695,899, of which 337,780 (48.54%) comprise empty parking space images and 358,119 (51.46%) comprise occupied space images. Figure 4b (empty sub image) and 4c (occupied sub image) show the segmented parking spaces.

## 4. Performance Evaluation of the System

### 4.1. Phase-I Using SCOPE with AlexNet

This pre-training transfer mechanism allows the CNN network’s parameters to be transferred from the natural imagery dataset to the car parking dataset. AlexNet is pre-trained on 1000 image classes and the last layers of AlexNet can be modified according to our dataset. The input layer of AlexNet only accepts RGB images with a size of 227 × 227 × 3, therefore, the images will be resized according to the input layer. Each layer in this network (e.g., convolutional layer, pooling layer) has a different filter size and has its own stride. According to the pre-trained AlexNet, every convolutional layer ends with a max pooling layer that will generate the greatest value based on a specified filter size. 

**Figure 5:**
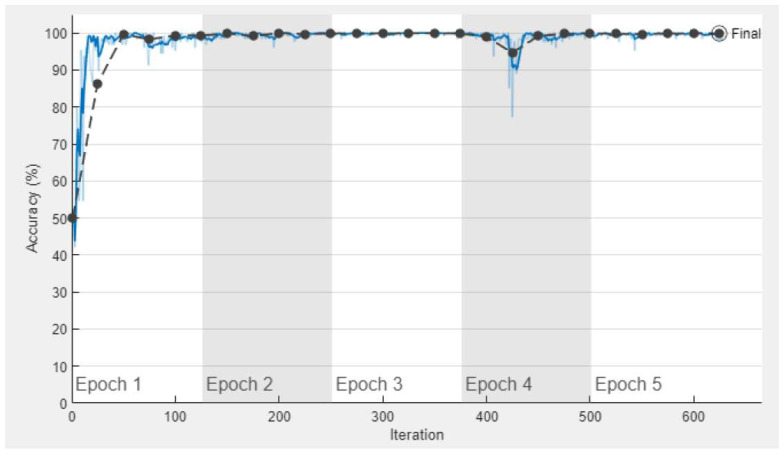
The training process of accuracy and validation of the proposed model with transfer learning (AlexNet). The light blue curve (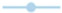) represents training accuracy and its smooth training accuracy curve is shown using the dark blue curve (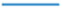). Further, the black curve (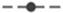) represents the training validation of the proposed model.

**Figure 6:**
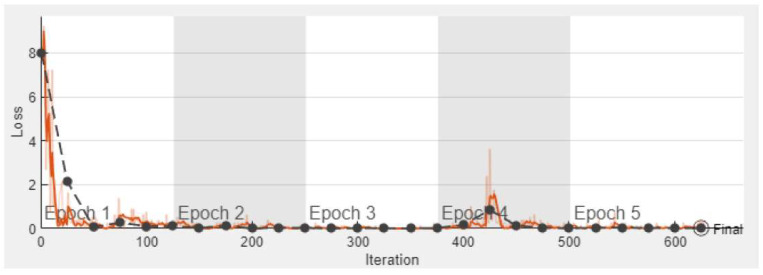
The training process of loss and validation of the proposed model with transfer learning (AlexNet). The light orange curve (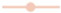) represents the number of losses and its smooth loss curve is shown using the dark orange line (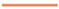). Lastly, the black curve (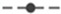) represents the loss validation of the proposed model.

Each convolutional layer visualizes the object features in the images, such as texture, angle, and the edge of the target images. Figure 5 shows the training graph using a customized AlexNet pre-trained network. This graph shows the accuracy of the training and validation dataset using five epochs with a 0.0001 learning rate. There are 625 total iterations and 125 iterations per epoch. Figure 6 shows the graph of the training process of the loss and validation of the dataset using five epochs with a 0.0001 learning rate. The number of iterations and losses are shown along the x-axis and y-axis, respectively. 

### 4.2. Phase-II Using SCOPE with YOLO

YOLO-V3 is also used to detect empty and occupied parking lots in real time. YOLO-V3 is a significantly better and faster than other techniques, such as R-CNN, and while R-CNN can be considered faster, it requires a lot of computations and repetition of processes [27]. On the other hand, YOLO-V3, as its name suggests, does all of its work in just one scan. In simple words, YOLO-V3 uses convolutional neural networks for object detection, and it is approximately six times faster than R-CNN. It can perform the following tasks:

- Detect multiple objects in an image.
- Predict multiple classes.
- Identify the locations of objects in the image.

**Figure 7:**
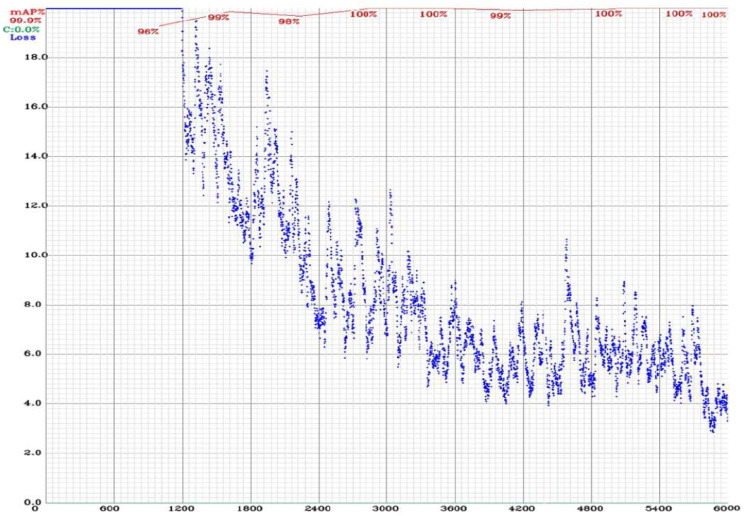
The training process of the proposed model using YOLOv3.

The YOLO training process is shown in Figure 7. Furthermore, during the training process, each image will be resized by a width of 416, and height of 426 is also required in the configuration file, which may be changed as needed. The 6000 iterations (max_batches) are identified to predict two classes (empty parking slots and occupied parking slots), and steps of 4800 and 5400 iterations (as per the policy) equal to 80% and 90% of the max_batches. The training configuration file uses a network size width = 416 and a height = 416, which means that every image will be resized to the network’s size during training and detection. 

The learning rate (learning_rate = 0.001) is a hyperparameter that adjusts and controls the weights of the network. The learning rate needs to be high at the beginning of the training process. Once you set the learning rate value, train the model, and wait for the learning rate to eventually decrease over time and enable the model to converge. The learning rate which decreases the policy is mentioned in the configuration file. The blue curve in Figure 7 shows the training loss, and the red curve shows the mean average precision (mAP), which is 99.9%. 

In this research, the performance of the proposed model is measured using accuracy, false negative rate (FNR), true positive rate (*TPR*), true negative rate (*TNR*), positive predictive value (*PPV*), negative predictive value (*NPV*), false positive rate (*FPR*), false discovery rate (*FDR*), and *F*1-*Score*.

The performance of the proposed model is evaluated based on the counts of validation records correctly and incorrectly predicted by the proposed trained model. The accuracy of the proposed model provides the information about how many images are correctly classified in the confusion matrix by using the trained proposed model, as shown in Equation (1).

(1) $$Accuracy=\frac{\left(\tau_{\rho }+\tau_{\nu }\right)\;}{N}\;$$

The error rate or miss rate or false negative rate (*FNR*) of the proposed model is calculated using Equation (2), and provides the information about how many images are incorrectly identified in the confusion matrix.

(2) $$Miss\;rate=\frac{\left(F_{\rho }+F_{\nu }\right)\;}{N}$$

The other metric of the measure of performance is sensitivity or recall or the true positive rate (*TPR*), and is calculated with Equation (3).

(3) $$Sensitivity=\frac{\tau_{\rho }}{\left(\tau_{\rho }+F_{\nu }\right)}$$

One more performance measure metric which is used is specificity or the true negative rate (*TNR*), and it is measured with Equation (4).

(4) $$Specificity=\frac{\tau_{\nu }}{\left(\tau_{\nu }+F_{\rho }\right)}$$

The precision or positive predictive value of the proposed model is measured with Equation (5).

(5) $$Precision=\frac{\tau_{\rho }}{\left(\tau_{\rho }+F_{\rho }\right)}$$

Equation (6) is used to find out the negative predictive value (*NPV*) of the proposed model.

(6) $$NPV=\frac{\tau_{\nu }}{\left(\tau_{\nu }+F_{\nu }\right)}$$

The false positive rate (*FPR*) of fallout of the proposed model is measured with Equation (7)

(7) $$FPR=\frac{F_{\rho }}{\left(\tau_{\nu }+F_{\rho }\right)}$$

Equation (8) represents the false discovery rate (*FDR*) of the proposed model.

(8) $$FDR=\frac{F_{\rho }}{\left(F_{\rho }+\tau_{\rho }\right)}$$

*F*1-*Score* is the import metric used to evaluate the proposed model. It is based on precision and recall, and is calculated by taking the geometric mean of recall and precision as shown in Equation (9).

(9) $$F1-Score=\frac{2*TPR*PPV}{TPR+PPV}$$

Table 2 shows the confusion matrix from the training phase of the proposed model used for automated parking lot detection prediction. These metrics are applied in the training and validation dataset, which is divided into 80% for the training dataset and 20% for the validation dataset. In this study, 80% of the training data set is used for building the proposed model and 20% of the dataset is used for measuring the proposed model’s accuracy. The results of the training and validation dataset in the form of a confusion matrix are shown in Table 2, Table 3 and Table 4. There are 20,000 randomly selected images from the parking lot dataset which are used for transfer learning with the use of a customized AlexNet network, in which 16,000 (80%) images are used for training and 4000 (20%) images are used to validate the training model, as shown in the respective tables.

Evaluation of the proposed model (transfer learning with AlexNet) using training and validation data, which employs various statistical measures for performance assessment as shown in Table 5.

Evaluation of the proposed model (transfer learning with YOLO) using training and validation data, which employs various statistical measures for performance assessment as shown in Table 6.

The description of the confusion matrix is shown in Table 1, and the results are shown below.

- True positive $\left(\tau_{\rho }\right)$ = 7991; the model accurately classified 7991 images in the empty lot class out of 8000 images.
- True negative $\tau_{\nu }$ = 7988; the model accurately classified 7988 images in the empty lot class out of 8000 images.
- False positive $\left(F_{\rho }\right)$ = 12; consequently, the model mistakenly identified 12 images of the occupied lot class as the empty lot class.
- False negative $\left(F_{\nu }\right)$ = 9; consequently, the model mistakenly identified 9 images of the empty lot class as the occupied lot class.

A total of 7991 and 7988 images were correctly identified as the empty lot and occupied lot classes, respectively.

Table 6 and Table 7 represent the proposed model’s measurements by using Equations (1)–(9). The accuracy, *FNR, TPR, TNR, PPV, NPV, FPR, FDR*, and *F*1-*Score* metrics of training and validation dataset of the proposed model are shown in Table 6. The rapid advancement of intelligent connected technologies and cellular vehicle-to-everything communication (C-V2X) presents new opportunities for addressing the challenges of connected automated vehicles (CAVs) at continuous signalized intersections, especially in the context of ecodriving [28].

## 5. Conclusions and Future Work

The emergence of smart cities has provided many challenges and requirements, including the autonomous data capturing ability and analytical provision for the end user and system to make decisions. For autonomous data capturing, IoT has become an ideal domain that provides the integration of multiple devices to capture dynamic data; therefore, the role of IoT, artificial intelligence, and analytics in the solutions related to smart cities is always prominent. As mentioned earlier, SCOPE is a model used for the management of the ecosystems of smart cities with cloud computing that gains autonomy using learning mechanisms and analytics. This paper has presented the data processing and learning components of SCOPE to validate two scenarios, i.e., by engaging AlexNet as the learning controller and by replacing AlexNet with YOLO-V3. The selected scenario investigated in this study is the identification of parking lot statuses, and, for this purpose, both models performed successfully as the learning controllers and provided significant results. The accuracy of AlexNet and YOLO models reached 99.87 and 99.89, respectively, while the comparison of the other models with previous results, which were significant, was also improved with the help of the proposed SCOPE model. It is important to note that the SCOPE model was evaluated on multiple other object identification scenarios for smart cities and has provided significant results in all scenarios. Future research should aim to explore the seamless integration of autonomous parking solutions with smart traffic management systems. This would involve real-time communication between vehicles and traffic infrastructure to optimize both parking and traffic flow within smart cities.

## Figures and Tables

**Table 1:** Year-wise different research findings.

Paper Title	Year	Research Focus	Key Finding
A survey of IoT-based smart parking systems in smart cities [21].	2019	IoT-based smart parking systems.	Provides a comprehensive overview of IoT-based parking systems, their components, and challenges.
Deep reinforcement learning for autonomous parking [22].	2020	Autonomous parking with deep reinforcement learning.	Discusses a deep reinforcement learning approach for autonomous parking.
Learning-based smart parking system [23].	2021	Intelligent detection of free parking slots.	Discusses convolution neural networks.
Autonomous detection of parking lots with multi-sensor data fusion using machine deep learning techniques [24].	2021	Deep convolutional neural network F-MTCNN.	Provides vision-based target detection and object classification.
Autonomous parking space detection for electric vehicles based on the improved YOLOV5-OBB algorithm [25].	2023	Receptive field block.	Discusses parking space detection and coordinate attention mechanism.

**Table 2:** Confusion matrix of the training of the proposed model during the prediction of automated parking lot detection.

	Expected Output (E_e_, E_o_)	O_e_ (Empty)	O_o_ (Occupied)	Total
Input	E_e_ (Empty)	7991	9	8000
	E_o_ (Occupied)	12	7988	8000
Total		8003	7997	16,000

**Table 3:** Confusion matrix of the validation of the proposed model using AlexNet during prediction of automated parking lot detection.

	Expected Output (E_e_, E_o_)	O_e_ (Empty)	O_o_ (Occupied)	Total
Input	E_e_ (Empty)	1997	3	2000
	E_o_ (Occupied)	4	1996	2000
Total		2001	1999	4000

**Table 4:** Confusion matrix of the validation of the proposed model using YOLO during the prediction of automated parking lot detection.

	Expected Output (E_e_, E_o_)	O_e_ (Empty)	O_o_ (Occupied)	Total
Input	E_e_ (Empty)	76,173	863	77,036
	E_o_ (Occupied)	2027	68,022	70,049
Total		78,200	68,885	147,085

**Table 5:** Performance evaluation of proposed model (transfer learning with AlexNet) using training and validation data with different statistical measures.

Results	Accuracy	FNR Miss Rate	TPR Sensitivity	TNR Specificity	PPV Precision	NPV	FPR	FDR	F1-Score
Training	0.9987 (99.87%)	0.0013 (0.13%)	0.9989 (99.89%)	0.9985 (99.85%)	0.9985 (99.85%)	0.9989 (99.89%)	0.0015 (0.15%)	0.0015 (0.15%)	0.9986 (99.87%)
Validation	0.9973 (99.73%)	0.0028 (0.28%)	0.9970 (99.70%)	0.9975 (99.75%)	0.9975 (99.75%)	0.9970 (99.70%)	0.00250 (0.25%)	0.00250 (0.25%)	0.9972 (99.73%)

**Table 6:** Performance evaluation of proposed model (transfer learning with YOLO) using validation data with different statistical measures.

Results	Accuracy	FNR Miss Rate	TPR Sensitivity	TNR Specificity	PPV Precision	NPV	FPR	FDR	F1-Score
Validation	0.9804 (98.04%)	0.0196 (1.96%)	0.9888 98.88%)	0.9711 (97.11%)	0.9741 (97.41%)	0.9875 (98.75%)	0.02894 (2.89%)	0.02592 (2.59%)	0.9814 (98.14%)

**Table 7:** The performance comparison of the proposed model with approaches in the literature.

Literature	Training		Validation	
	Accuracy (%)	Miss Rate (%)	Accuracy (%)	Miss Rate (%)
Fabian (2013) [22]	96.40	3.60	96.2	3.80
Amato et al. (2018) [23]	96.36	3.64	96.1	3.90
Kashif et al. (2020) [24]	97.60	2.40	96.6	3.40
Proposed model (YOLO)	99.89	0.11	98.04	1.96
Proposed model (AlexNet)	99.87	0.13	99.73	0.27

## Data Availability

The data used in this paper can be requested from the corresponding authors upon request.
